# Caerin 1 Antimicrobial Peptides that Inhibit HIV and *Neisseria* May Spare Protective Lactobacilli

**DOI:** 10.3390/antibiotics9100661

**Published:** 2020-09-30

**Authors:** Louise A. Rollins-Smith, Patricia B. Smith, Anna M. Ledeczi, Julia M. Rowe, Laura K. Reinert

**Affiliations:** Departments of Pathology, Microbiology and Immunology and of Pediatrics, Vanderbilt University School of Medicine and Department of Biological Sciences, Vanderbilt University, Nashville, TN 37232, USA; patricia.mcdeavitt@gmail.com (P.B.S.); aml2312@columbia.edu (A.M.L.); rowejm@etsu.edu (J.M.R.); laura.reinert@vumc.org (L.K.R.)

**Keywords:** amphibian antimicrobial peptide, caerin peptide, human immunodeficiency virus (HIV), *Lactobacillus crispatus*, *Lactobacillus rhamnosus*, *Neisseria lactamica*

## Abstract

Although acquired immunodeficiency syndrome (AIDS) caused by the human immunodeficiency virus (HIV) is a manageable disease for many, it is still a source of significant morbidity and economic hardship for many others. The predominant mode of transmission of HIV/AIDS is sexual intercourse, and measures to reduce transmission are needed. Previously, we showed that caerin 1 antimicrobial peptides (AMPs) originally derived from Australian amphibians inhibited in vitro transmission of HIV at relatively low concentrations and had low toxicity for T cells and an endocervical cell line. The use of AMPs as part of microbicidal formulations would expose the vaginal microbiome to these agents and cause potential harm to protective lactobacilli. Here, we tested the effects of caerin 1 peptides and their analogs on the viability of two species of common vaginal lactobacilli (*Lactobacillus rhamnosus* and *Lactobacillus crispatus*). Several candidate peptides had limited toxicity for the lactobacilli at a range of concentrations that would inhibit HIV. Three AMPs were also tested for their ability to inhibit growth of *Neisseria lactamica*, a close relative of the sexually transmissible *Neisseria gonorrhoeae. Neisseria lactamica* was significantly more sensitive to the AMPs than the lactobacilli. Thus, several candidate AMPs have the capacity to inhibit HIV and possible *N. gonorrhoeae* transmission at concentrations that are significantly less harmful to the resident lactobacilli.

## 1. Introduction

According to the World Health Organization, there were about 38 million people living with HIV/AIDS, 1.7 million new HIV infections, and 690,000 HIV-related deaths in 2019 [1]. Thus, this disease continues to cause significant worldwide morbidity and mortality. Although AIDS has become a manageable disease in resource-rich countries, it remains a significant health and wealth burden everywhere, especially in resource-poor regions [2]. A long-term but elusive research goal has been the development of a vaccine. In the absence of an effective vaccine, other prevention and control measures have been sought. Although antiretroviral therapy has become an effective method to both control the disease and stop transmission, new infections still occur because many who are infected either do not know they are infected or do not have access to pre-exposure prophylaxis. One possible method to reduce transmission is development of low-cost antiviral microbicides that could be introduced into the vaginal or rectal space. Several studies have shown reduced transmission with the use of the small molecule inhibitors tenofovir [3] or dapivirine [4,5] that target viral reverse transcription (reviewed in [6]). However, effectiveness was reduced in the presence of inflammation [7]. Disruption of the vaginal microbiome, especially the absence of lactobacilli, has been associated with increased risk of HIV infection [8,9]. AMPs have been proposed as possible alternative microbicides for prevention of HIV transmission [10,11]. They are naturally present in human mucosal secretions and reach very high concentrations in the natural mucosal secretions of amphibians. Previously, we showed that a number of amphibian caerin 1 peptides isolated from Australian frogs inhibited HIV transmission in vitro and transfer of virions from dendritic cells to T cells with low toxicity against T cells and endocervical epithelial cell lines [10,11]. Because the use of AMPs as microbicides would introduce them to the vaginal microbial community, we examined the effects of a set of caerin 1 peptides and their analogs on the viability of two species of lactobacilli (*Lactobacillus rhamnosus* and *Lactobacillus crispatus*). Both of these species have been reported to have protective effects in the vaginal environment to prevent HIV transmission [12,13]. Here, we show that most of the peptides tested had limited toxicity for both species of lactobacilli at concentrations that have been shown to inhibit HIV. Three peptides were also tested for their capacity to inhibit growth of *Neisseria lactamica*, a surrogate for the pathogenic *Neisseria gonorrhoeae.* Two were highly effective at inhibiting the growth of *N. lactamica,* suggesting they could also protect against gonorrhea.

## 2. Results

### 2.1. Effects of Caerin 1 Peptides on Growth of Lactobacillus rhamnosus

The caerin-1 peptides tested for their effects on growth of *Lactobacillus rhamnosus* are shown in Table 1. Caerin 1.1 was tested previously for HIV inhibition, but it was not tested for activity against lactobacilli in these studies because it was less effective in the inhibition of HIV transmission than other caerin family peptides [10,11]. Its sequence is shown here for comparison with the synthetically modified caerin 1.1 peptide. Amino acid modifications to the caerin 1.1 and to the caerin 1.9 peptides are shown in bold type with gray shading. All of the peptides studied have many similarities to the more thoroughly studied caerin 1.1. Like, caerin 1.1, caerin 1.2, 1.9, 1.10, and 1.20 are wide-spectrum anti-bacterial agents [14,15]. In a membrane-like environment, they adopt a hinged amphipathic structure with two alpha-helices on either side of the hinge [16,17,18]. We included caerin 1.3 and caerin 1.4 as control peptides because they had previously been shown to have reduced antibacterial activity. For example, in the naturally occurring caerin 1.3, a lysine (K) in caerin 1.1 at position 11 is replaced by glutamine (Q) leading in caerin 1.3 to a reduction of activity against most of the bacteria tested [16]. Caerin 1.1 mod 7 contains a replacement of a glutamic acid residue and a histidine residue by two alanine residues. The histidine at position 24 is thought to provide a positive charge to enable binding to negative charges on a bacterial membrane. Thus, the replacement with alanine could affect the charged state of the peptide at the amino terminal end. The alanine substitutions may also alter helix formation [17]. Caerin 1.9 sm has a single amino acid change from the sequence of caerin 1.9. A valine is replaced by a leucine at position 13. This would be considered a conservative substitution that would likely not change the activity of the peptide.

**Figure 1:**
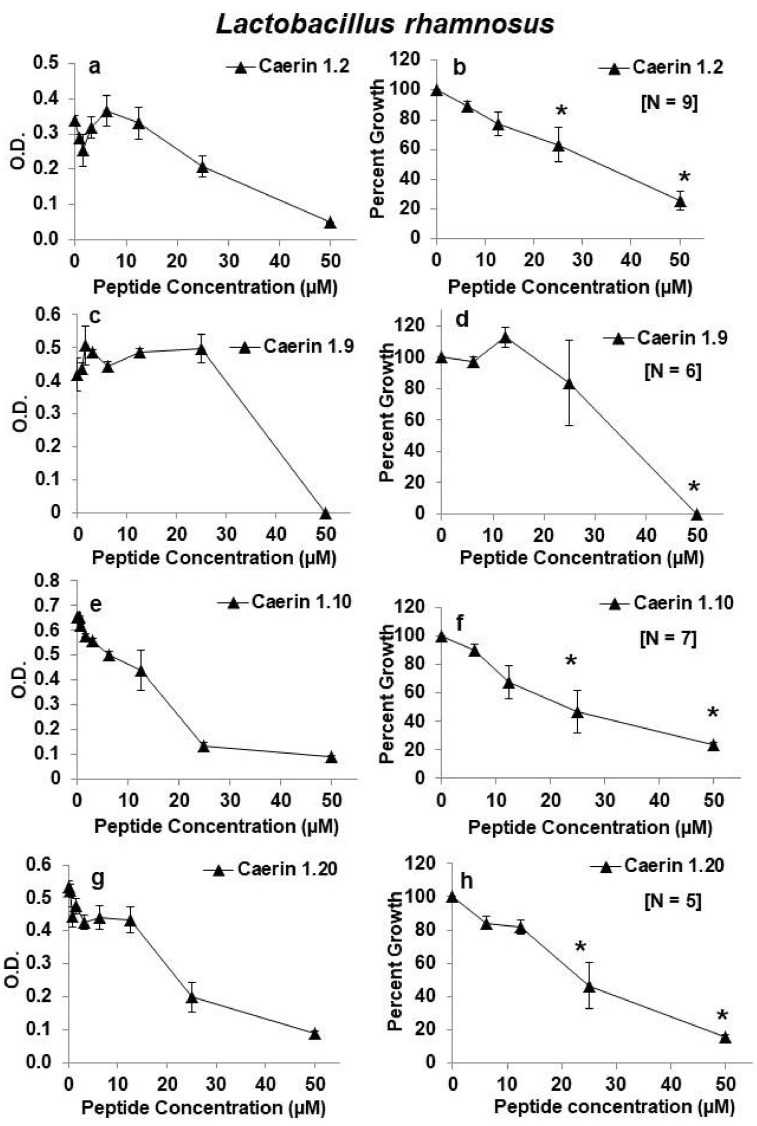
Effects of caerin 1 peptides on growth of *Lactobacillus rhamnosus*. (**a**,**b**) Caerin 1.2; (**c**,**d**) Caerin 1.9; (**e**,**f**) Caerin 1.10; (**g**,**h**) Caerin 1.20. Panels on the left show growth as changes in optical density (O.D.) at 630 nm for one representative experiment testing the peptides at increasing 2-fold concentrations from 0.4 or 0.8 to 50 µM. Panels on the right show the average as percent growth in comparison with the positive (no peptide) control set at 100% for replicate experiments for the range of concentrations from 6.25–50 µM. The number of replicate experiments for each peptide is shown in brackets within the panel. * Significantly reduced growth in comparison with the positive control, *p* < 0.01 by one-way ANOVA with Tukey’s post hoc test.

**Figure 2:**
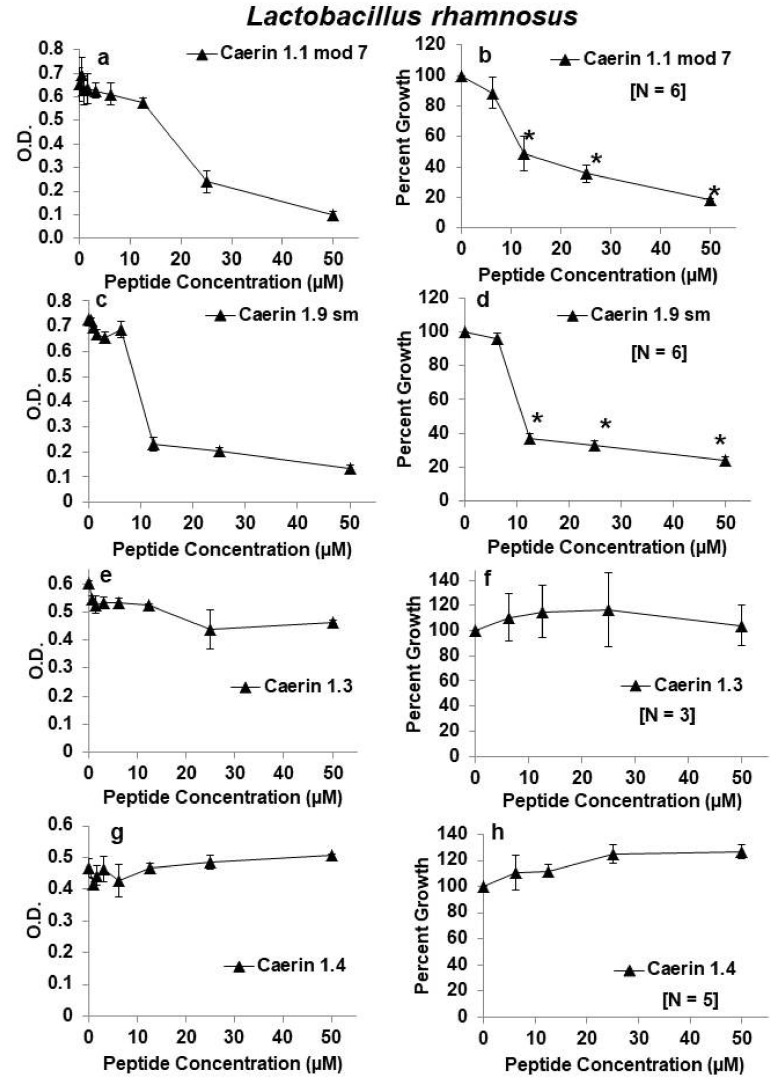
Effects of caerin 1 peptides on growth of *Lactobacillus rhamnosus.* (**a**,**b**) Caerin 1.1 mod 7; (**c**,**d**) Caerin 1.9 sm; (**e**,**f**) Caerin 1.3; (**g**,**h**) Caerin 1.4. Panels on the left show growth as changes in optical density (O.D.) at 630 nm for one representative experiment testing the peptides at increasing 2-fold concentrations from 0.4 or 0.8 to 50 µM. Panels on the right show the average as percent growth in comparison with the positive (no peptide) control set at 100% for replicate experiments for the range of concentrations from 6.25–50 µM. The number of replicate experiments for each peptide is shown in brackets within the panel. * Significantly reduced growth in comparison with the positive control, *p* < 0.01 by one-way ANOVA with Tukey’s post hoc test.

Our previous studies suggested that four of the caerin peptides (caerin 1.2, caerin 1.9, caerin 1.10, and caerin 1.20) were very effective inhibitors of HIV with limited toxicity to T lymphocytes or endocervical cell lines [10,11]. Thus, we first tested these four peptides for effects on growth of *L. rhamnosus*. Although each peptide significantly inhibited growth at a concentration of 50 µm, viability as measured by 24-h growth of *L. rhamnosus* remained quite high over a range of concentrations from 0.4–25 µM for each of these four peptides. Of these first four peptides examined, caerin 1.9 appeared to have the least antibacterial activity against *L. rhamnosus* with average bacterial growth at about 83% at a concentration of 25 µm. At 25 µM, each of the other three peptides reduced *L. rhamnosus* growth by about 40–55% (Figure 1). Two other peptides (caerin 1.1 mod 7 and caerin 1.9 sm) inhibited growth to a greater extent than the previous four, and two others (caerin 1.3 and 1.4) showed almost no inhibition at any dose tested (Figure 2).

### 2.2. Effects of Caerin 1 Peptides on Growth of Lactobacillus crispatus

**Figure 3:**
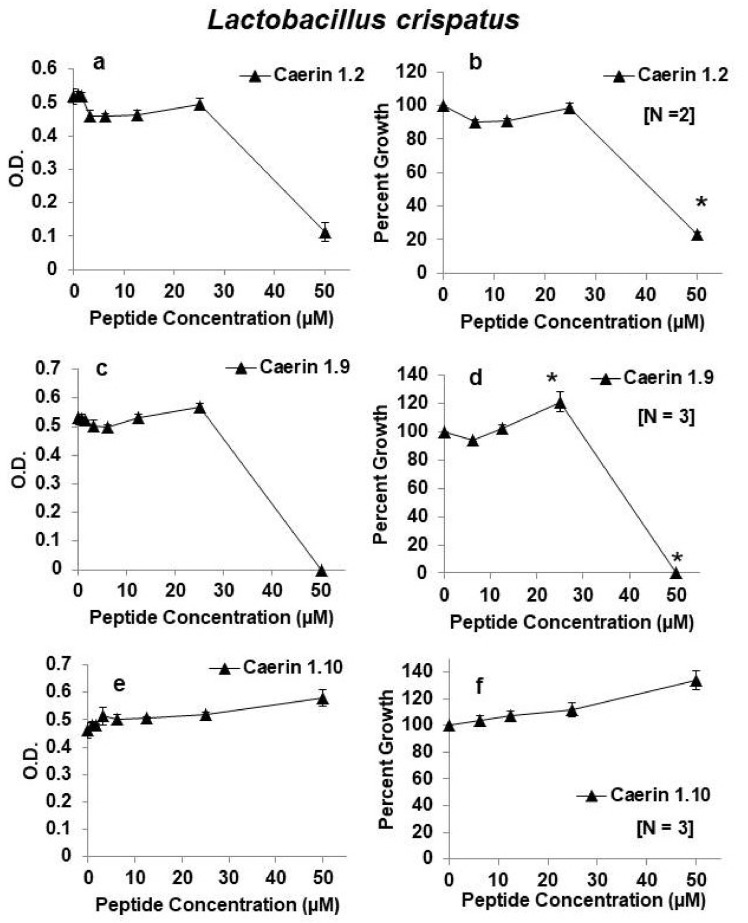
Effects of caerin 1 peptides on growth of *Lactobacillus crispatus.* (**a**,**b**) caerin 1.2; (**c**,**d**) caerin 1.9; (**e**,**f**) caerin 1.10. Panels on the left show growth as changes in optical density (O.D.) at 630 nm for one representative experiment testing the peptides at increasing 2-fold concentrations from 0.4 or 0.8 to 50 µM. Panels on the right show the average as percent growth in comparison with the positive (no peptide) control set at 100% for replicate experiments for the range of concentrations from 6.25–50 µM. The number of replicate experiments for each peptide is shown in brackets within the panel. * Significantly different growth in comparison with the positive control, *p* < 0.01 by one-way ANOVA with Tukey’s post hoc test.

**Figure 4:**
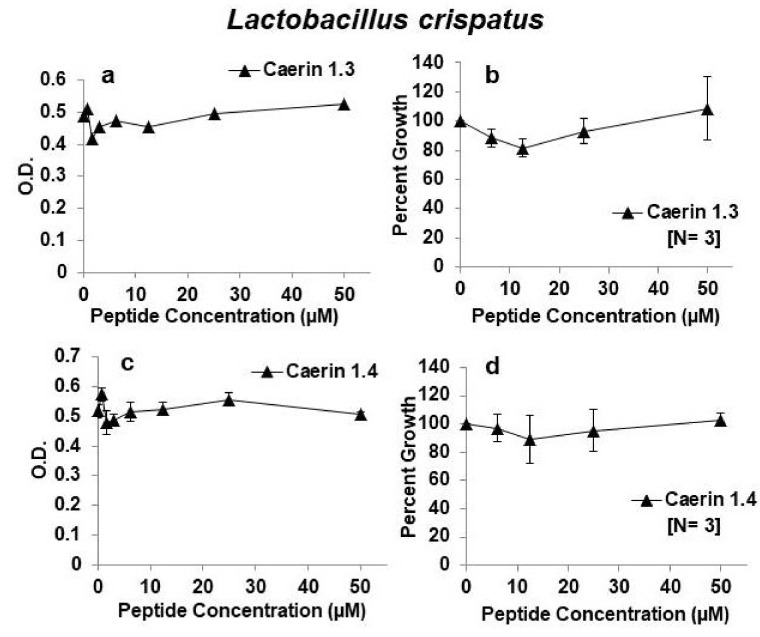
Effects of caerin 1 peptides on growth of *Lactobacillus crispatus*. (**a**,**b**) caerin 1.3; (**c**,**d**) caerin 1.4. Panels on the left show growth as changes in optical density (O.D.) at 630 nm for one representative experiment testing the peptides at increasing 2-fold concentrations from 0.4 or 0.8 to 50 µM. Panels on the right show the average as percent growth in comparison with the positive (no peptide) control set at 100% for replicate experiments for the range of concentrations from 6.25–50 µM. The number of replicate experiments for each peptide is shown in brackets within the panel.

Because caerin 1.2, caerin 1.9, and caerin 1.10 appeared to be the most effective inhibitory peptides against HIV and least inhibitory towards *L. rhamnosus*, we next tested their effects on the growth of *L. crispatus.* Both caerin 1.2 and caerin 1.9 had a similar profile of inhibition of *L. crispatus* as that of *L. rhamnosus*. That is, they were inhibitory at the highest concentration tested (50 µM) but showed almost no inhibition at concentrations of 25 µM or less. Furthermore, in three independent experiments, caerin 1.10 showed no inhibition of *L. crispatus* growth even at the highest concentration of 50 µM (Figure 3). These experiments suggest that *L. crispatus* is equally or more resistant to growth inhibition by these three peptides as *L. rhamnosus*. We also tested the effects of caerin 1.3 and caerin 1.4 on the growth of *L. crispatus*. Just like growth of *L. rhamnosus*, growth of *L. crispatus* was largely unaffected by these two peptides (Figure 4).

### 2.3. Comparison of the Effects of Caerin 1 Peptides on HIV Infection and Growth of Lactobacilli

**Figure 5:**
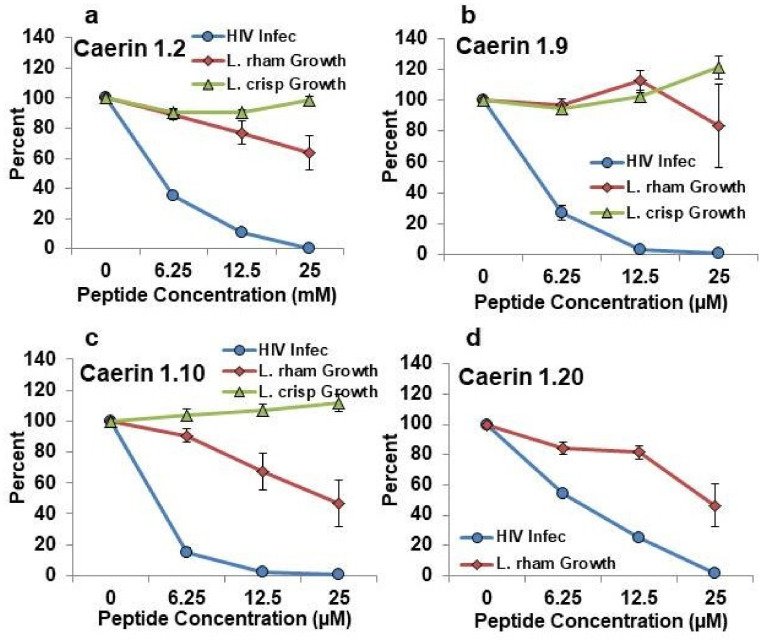
Comparison of the effects of caerin 1 peptides on percent of HIV infection of target T cells (blue) with percent growth of *Lactobacillus rhamnosus* (red) and *Lactobacillus crispatus* (green). (**a**) caerin 1.2; (**b**) caerin 1.9; (**c**) caerin 1.10; (**d**) caerin 1.20. HIV percent infection was previously published (11) and average percent growth of the lactobacilli in the presence of each peptide is new for this study and presented above in Figure 1, Figure 2 and Figure 3.

**Figure 6:**
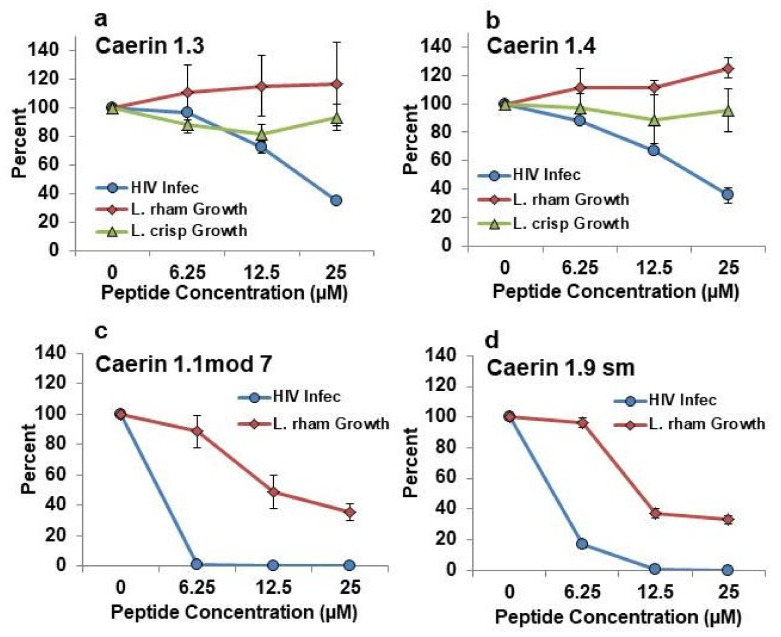
Comparison of the effects of caerin 1 peptides on percent of HIV infection of target T cells (blue) with percent growth of *Lactobacillus rhamnosus* (red) and *Lactobacillus crispatus* (green). (**a**) caerin 1.3; (**b**) caerin 1.4; (**c**) caerin 1.1 mod 7; (**d**) caerin 1.9 sm. HIV percent infection was previously published [11] and average percent growth of the lactobacilli in the presence of each peptide is new for this study and presented above in Figure 1, Figure 2, Figure 3 and Figure 4.

In order to be useful as a microbicide for prevention of HIV infection, a given peptide should be highly effective in prevention of HIV infection yet minimally harmful to the protective lactobacilli. To evaluate both parameters at once, we plotted the patterns of inhibition of HIV infection from a previous publication [11] on the same axes as the new data for effects on *L. rhamnosus* and *L. crispatus* growth. These data are shown for caerin 1.2, caerin 1.9, caerin 1.10, and caerin 1.20 in Figure 5 and for caerin 1.3, caerin 1.4, caerin 1.1 mod 7, and caerin 1.9 sm in Figure 6. Among all of the peptides tested, caerin 1.9 showed the best profile of inhibition of HIV over the range of concentrations of 6.25–25 µM with the least apparent inhibition of growth of both tested lactobacilli. Caerin 1.2 and caerin 1.10 were also effective inhibitors of HIV infection, but each inhibited one of the species of lactobacilli somewhat more than caerin 1.9. Caerin 1.20 was slightly less effective in the inhibition of HIV and slightly more inhibitory against *L. rhamnosus* than caerin 1.9 (Figure 5). Caerin 1.1 mod 7 and caerin 1.9 sm showed equal or greater inhibition of HIV infection than caerin 1.9, but both inhibited the growth of *L. rhamnosus* more than caerin 1.9. Caerin 1.3 and caerin 1.4 inhibited HIV less than caerin 1.9 and showed very limited inhibition of both species of lactobacilli (Figure 6).

### 2.4. Effects of Caerin 1 Peptides on Growth of Neisseria lactamica

**Figure 7:**
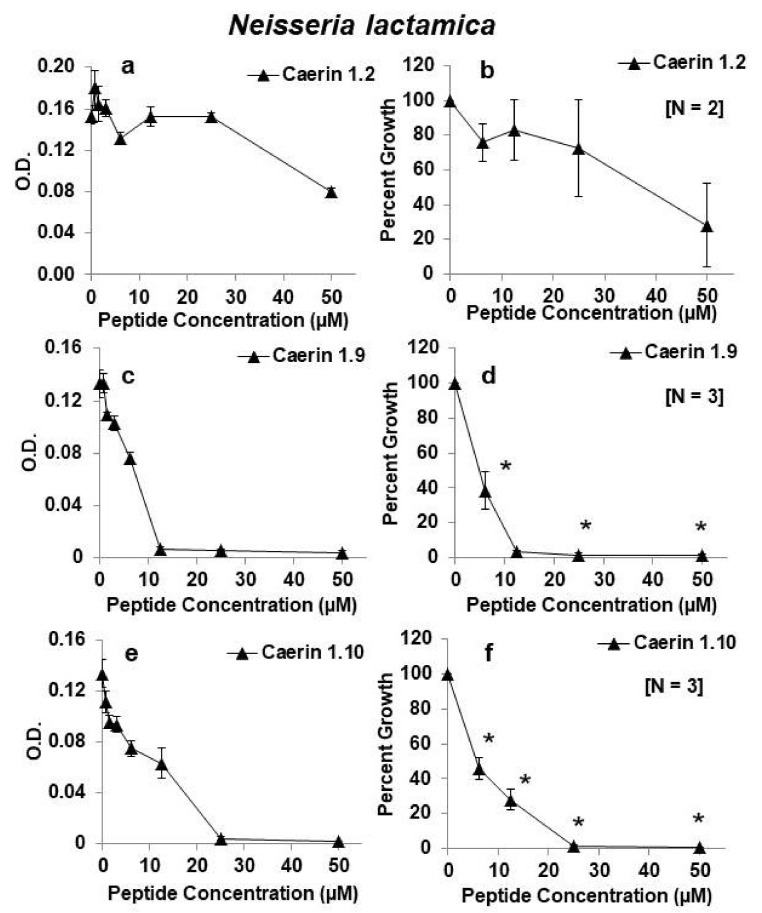
Effects of caerin 1 peptides on growth of *Neisseria lactamica.* (**a**,**b**) caerin 1.2; (**c**,**d**) caerin 1.9; (**e**,**f**) caerin 1.10. Panels on the left show growth as changes in optical density (O.D.) at 630 nm for one representative experiment testing the peptides at increasing 2-fold concentrations from 0.8 to 50 µM. Panels on the right show the average as percent growth in comparison with the positive (no peptide) control set at 100% for replicate experiments for the range of concentrations from 6.25–50 µM. The number of replicate experiments for each peptide is shown in brackets within the panel. * Significantly different growth in comparison with the positive control, *p* < 0.01 by one-way ANOVA with Tukey’s post hoc test.

Antimicrobial peptides applied for antiretroviral protection in the vagina or rectum could also play a role in protection from other sexually transmitted organisms such as *Neisseria gonorrhoeae*, the causative agent of gonorrhea. *N. lactamica* is in the same genus as *N. gonorrhoeae*, and both are Gram negative. While they are distinct species, it has been shown that the species within the *Neisseria* genus share the majority of their genes, including 93% of the genes previously thought to influence their virulence and pathogenicity [19]. Thus, *N. lactamica* is a good nonpathogenic model organism to study the effects of the microbicides on the closely related *N. gonorrhoeae*. To that end, we examined the effects of caerin 1.2, caerin 1.9, and caerin 1.10 on growth of *N. lactamica.* In contrast to their effects on the lactobacilli, both caerin 1.9 and 1.10 were highly effective in the inhibition of growth of *N. lactamica* at concentrations as low as 6.2 µM. Although we only tested caerin 1.2 twice, it appeared to be less effective against *N. lactamica* than caerin 1.9 and caerin 1.10 (Figure 7).

## 3. Discussion

The importance of lactobacilli and their capacity to produce antimicrobial substances and maintain low pH in the human vagina for protection from bacterial vaginosis has long been appreciated (reviewed in [20]). The healthy vaginal community of most ethnic groups in North America is dominated by several species of lactobacilli including *Lactobacillus crispatus* [21]. Disruptions in the vaginal microbiome, such as in bacterial vaginosis and sexually transmitted infections, such as gonorrhea, have been associated with depleted lactobacilli communities, greater inflammation, and less protection from HIV infection [8,13,22,23,24]. Furthermore, inflammation due to genital infections appeared to interfere with the effectiveness of the antiretroviral tenofovir [7]. In a large study of young South African women who were initially HIV seronegative, those with *L. crispatus*-dominated vaginal microbiota were significantly less likely to become infected with HIV. Depleted *L. crispatus* and diverse anaerobes, such as *Prevotella*, *Sneathia*, and other anaerobes, were associated with greater HIV infection and greater numbers of mucosal CD4^+^ T cells, the target of HIV [13]. One of the likely protective mechanisms is improved barrier function when the lactobacilli attach to the epithelium of the female genital tract. Recent studies suggest that both estrogen and lactobacilli, including possible probiotic strains such as *L. rhamnosus*, improve barrier function [12]. Thus, the importance of lactobacilli in protection from HIV remains an important consideration in the design of possible anti-HIV microbicides. Here, we showed that several caerin 1 AMPs that can inhibit HIV infection have few or no deleterious effects on two species of lactobacilli at a range of concentrations at which HIV is inhibited. Among the eight peptides tested in this study, caerin 1.9 emerged as a highly effective anti-HIV agent with limited toxicity to *L. rhamnosus* and *L. crispatus*. Although several of the other peptides have very similar or almost identical sequences as caerin 1.9, they were less effective against HIV or showed greater inhibition of resident epithelial cells or lactobacilli ([11] and this study). For example, caerin 1.9 sm has one amino acid change from the sequence of caerin 1.9. A valine is replaced by a leucine at position 13. Although this would be considered to be a conservative substitution, this study showed that caerin 1.9 sm inhibited growth of *L. rhamnosus* more than the natural caerin 1.9. Without three-dimensional analysis of different peptide interactions with the HIV envelope or with the cell wall of the lactobacilli, it is difficult to predict how a given peptide will interact with the candidate microbe in a physiological system. In my view, that is the value of this kind of comparative testing of natural caerin peptides and their modified analogs to find the best potential microbicide.

Caerin 1.9 was also highly effective in the inhibition of growth of *Neisseria lactamica*, a surrogate for the study of *Neisseria gonorrhoeae*. While these species are distinct, they share many genes [19]. The caerin family peptides are thought to interact with bacterial cell membranes by a carpet-like mechanism whereby the peptides aggregate and orient themselves parallel to the membrane in a sheet-like arrangement followed by disruption of the bacterial cell membranes [16]. Thus, they are likely to act in the same way to inhibit growth of both species of *Neisseria*.

Lactobacilli themselves produce their own antimicrobial peptides designated bacteriocins ([25,26] reviewed in [27]). Thus, the vaginal microenvironment may be a suitable environment for introduction of other AMPs. They could be introduced as soluble peptides in a gel or one can envision designing probiotic lactobacilli that could colonize the female vagina and produce protective levels of natural AMPs or other antivirals along with AMPs to inhibit HIV and other viral or bacterial sexually transmitted diseases. A number of studies using recombinant bacteria to express antiviral activity against HIV or the simian-human immunodeficiency virus (SHIV) hold great promise [28,29,30].

## 4. Materials and Methods

### 4.1. Peptides

The peptides were synthesized by Chiron Mimotopes (Clayton, Victoria, Australia) or Genscript Corporation (Piscataway, NJ, USA) using L-amino acids and standard 9-fluorenylmethoxycarbonyl (Fmoc) chemistry. Most of them were kindly provided as a gift by Professor John Bowie, Adelaide University. Peptides were prepared at 1 mM concentrations in HPLC-grade water. Mixtures were filter sterilized and stored at −20 °C. Masses, sequences, and species of origin are listed for each peptide in Table 1. Mass spectrometry was used to determine if peptides were intact to ensure the validity of assays performed as previously described [31]. Briefly, the peptides were resuspended at a concentration of 1 mg/mL in HPLC grade water, and a mixture containing 0.6 µL of resuspended peptides and 0.6 µL of α-Cyano-4-hydroxycinnamic acid matrix (CHCA) (Sigma, St. Louis, MO, USA) was spotted onto the target plate and air-dried. MALDI-TOF MS was performed using the Bruker Daltronics Ultraflex III time-of flight mass spectrometer (Bruker, Billerica, MA, USA) operated in reflector, delayed extraction, and positive ion mode. The instrument was calibrated using a mixture of standard peptides including bradykinin fragment 1-7 (*m/z* 757.3996), human angiotensin II (*m/z* 1046.5423), P_14_R synthetic peptide (*m/z* 1533.8582), ACTH (*m/z* 24565.1989), and bovine oxidized insulin chain B (*m/z* 3494.65113). Automated data acquisition was performed by averaging 250 laser shots. Peaks observed were compared to the known average peptide masses, listed in Table 1. If a peptide was degraded following storage in the laboratory, it was not used in this research.

### 4.2. Bacteria and Bacterial Growth Assays

Bacterial strains were obtained from the American Type Culture Collection (*Lactobacillus crispatus*, ATCC^®^ 33197™; *Lactobacillus rhamnosus,* ATCC^®^ 39595™; *Neisseria lactamica* (ATCC^®^ 23970™). Growth assays were conducted to determine the concentrations at which each peptide inhibited the growth of the three bacterial species. The *Lactobacillus* bacteria were grown in de Man, Rogosa, and Sharpe (MRS) broth and the *N. lactamica* in Fastidious Broth [32,33]. Bacterial cells were cultured in broth for 24 to 48 h until they reached an optical density of about 0.1 O.D. units at 630 nm and diluted to achieve a concentration of 10^6^ cells per mL. The peptides were serially diluted with sterile water to reach peptide concentrations of 0.4, 0.8, 1.6, 3.12, 6.25, 12.5, 25, and 50 µM final concentrations in culture. The bacterial cells at 10^6^ cells/mL and the peptides were plated in triplicate in a 96-well plate. Included was a negative control for growth (three to five replicates of bacteria plus penicillin-streptomycin), a positive control for growth (three to five replicates of bacteria plus water), and a blank (three to five replicates of broth plus water) to ensure the broth was not contaminated. Bacterial cells were plated in 50 µL of broth, and each dilution of peptides was added in a volume of 50 µL to achieve a total volume of 100 µL. For the positive control, 50 µL of sterile water was added to 50 µL of cells in broth. For the negative control, 50 µL of the antibiotic solution was added to 50 µL of cells in broth. The O.D. of each well was recorded before and after a 24-h incubation period at 37 °C using a Biotek ELx808 reader (Biotek, Winooski, VT, USA). Data for representative experiments are expressed as average O.D. ± standard error of the mean at each peptide concentration. To express data from replicate experiments, the average as percent growth in comparison with the positive (no peptide) control set at 100% for the range of concentrations from 6.25–50 µM is shown. Significantly different growth in comparison with the positive control was determined by one-way analysis of variance (ANOVA) with Tukey’s post hoc test. A *p*-value of less than 0.05 was considered significant.

## 5. Conclusions

This study lends additional support to our two previous studies of caerin 1 peptides as possible microbicides to limit sexual transmission of HIV [10,11]. In each of the previous studies and this study, caerin 1.9 emerged as the best peptide for excellent inhibition of HIV transmission with limited deleterious effects on T lymphocytes, an endocervical cell line, and two species of common lactobacilli. Although preliminary, we also showed that caerin 1.9 could inhibit growth of *Neisseria lactamica*, which shares many characteristics with pathogenic *Neisseria gonorrhoeae*. Thus, a microbicide employed to limit HIV transmission might also limit transmission of other pathogenic vaginal bacteria. 

## Figures and Tables

**Table 1:** Caerin 1 Antimicrobial Peptides.

Peptide	Species of Origin	Sequence	Mass
Caerin 1.1	*Litoria caerulea*	GLLSVLGSVAKHVLPHVVPVIAEHL-NH_2_	2582
Caerin 1.1 mod 7	synthetic	GLLSVLGSVAKHVLPHVVPVIAAAL-NH_2_	2458
Caerin 1.2	*L. caerulea*	GLLGVLGSVAKHVLPHVVPVIAEHL-NH_2_	2552
Caerin 1.3	*L. caerulea*	GLLSVLGSVAQHVLPHVVPVIAEHL-NH_2_	2582
Caerin 1.4	*L. caerulea, L. gilleni*	GLLSSLSSVAKHVLPHVVPVIAEHL-NH_2_	2600
Caerin 1.9	*L. chloris*	GLFGVLGSIAKHVLPHVVPVIAEKL-NH_2_	2591
Caerin 1.9 sm	synthetic	GLFGVLGSIAKHLLPHVVPVIAEKL-NH_2_	2605
Caerin 1.10	*L. splendida*	GLLSVLGSVAKHVLPHVVPVIAEKL-NH_2_	2573
Caerin 1.20	*L. caerulea/L. splendida*	GLFGILGSVAKHVLPHVIPVVAEHL-NH_2_	2600

## References

[1] World Health Organization. www.who.int.

[2] Gona P.N., Gona C.M., Ballout S., Rao S.R., Kimokoti R., Mapoma C.C., Mokdad A.H. (2020). Burden and changes in HIV/AIDS morbidity and mortality in Southern Africa Development Community Countries, 1990–2017. BMC Public Health.

[3] Abdool Karim Q., Abdool Karim S.S., Frohlich J.A., Grobler A.C., Baxter C., Mansoor L.E., Kharsany A.B.M., Sibeko S., Mlisana K.P., Omar Z. (2010). Effectiveness and safety of tenofovir gel, an antiretroviral microbicide, for the prevention of HIV infection in women. Science.

[4] Baeten J.M., Palanee-Phillips T., Brown E.R., Schwartz K., Soto-Torres L.E., Govender V., Jeenarain N., Gaffoor A., Martinson F., Makanani B. (2016). Use of a vaginal ring containing dapivirine for HIV-1 prevention in women. N. Engl. J. Med..

[5] Nel A., van Niekerk N., Kapiga S., Bekker L.G., Gama C., Gill K., Kamali A., Kotze P., Louw C., Mabude Z. (2016). Safety and efficacy of a dapivirine vaginal ring for HIV prevention in women. N. Engl. J. Med..

[6] Cohen M.S., Council O.D., Chen J.S. (2019). Sexually transmitted infections and HIV in the era of antiretroviral treatment and prevention: The biologic basis for epidemiologic synergy. J. Int. AIDS Soc..

[7] McKinnon L.R., Liebenberg L.J., Yende-Zuma N., Archary S., Ngcapu S., Sivro A., Nagelkerke N., Lerma G.G., Kashuba A.D., Masson L. (2018). Genital inflammation undermines the effectiveness of tenofovir gel in preventing HIV acquisition in women. Nat. Med..

[8] Martin H.L., Richardson B.A., Nyange P.M., Lavreys L., Hillier S.L., Chohan B., Mandaliya K., Ndinya-Achola J.L., Bwayo J., Kreiss J. (1999). Vaginal lactobacilli, microbial flora, and risk of human immunodeficiency virus type 1 and sexually transmitted disease acquisition. J. Infect. Dis..

[9] McClelland R.S., Lingappa J.R., Srinivasan S., Kinuthia J., John-Steward G.S., Jaoko W., Richardson G.A., Yuhas K., Fiedler T.L., Mandaliya K.N. (2018). Key vaginal bacteria associated with increased risk of HIV acquisition in African women: A nested case-control study. Lancet Infect Dis..

[10] VanCompernolle S.E., Taylor R.J., Oswald-Richter K., Jiang J., Youree B.E., Bowie J.H., Tyler M.J., Conlon J.M., Wade D., KewalRamani V.N. (2005). Amphibian antimicrobial skin peptides potently inhibit HIV infection and transfer of virus from dendritic cells to T cells. J. Virol..

[11] VanCompernolle S., Smith P.B., Bowie J.H., Tyler M.J., Unutmaz D., Rollins-Smith L.A. (2015). Inhibition of HIV infection by caerin 1 antimicrobial peptides. Peptides.

[12] Dizzell A., Nazli A., Reid G., Kaushic C. (2019). Protective effect of probiotic bacteria and estrogen in preventing HIV-1-mediated impairment of epithelial barrier integrity in female genital tract. Cells.

[13] Gosmann C., Anahtar M.N., Handley S.A., Farcasanu M., Abu-Ali G., Bowman B.A., Padavattan N., Desai C., Droit L., Moodley A. (2017). Lactobacillus-deficient cervicovaginal bacterial communities are associated with increased HIV acquisition in young South African women. Immunity.

[14] Steinborner S.T., Currie G.J., Bowie J.H., Wallace J.C., Tyler M.J. (1998). New antibiotic caerin 1 peptides from the skin secretion of the Australian tree frog *Litoria chloris*. Comparison of the activities of the caerin 1 peptides from the genus Litoria. J. Peptide Res..

[15] Pukala T.L., Bertozzi T., Donnellan S.C., Bowie J.H., Surinya-Johnson K.H., Liu Y., Jackway R.J., Doyle J.R., Llewellyn L.E., Tyler M.J. (2006). Host-defence peptide profiles of the skin secretions of interspecific hybrid tree frogs and their parents, female *Litoria splendida* and male *Litoria caerulea*. FEBS J..

[16] Wong H., Bowie J.H., Carver J.A. (1997). The solution structure and activity of caerin 1.1, an antimicrobial peptide from the Australian green tree frog, Litoria splendida. Eur. J. Biochem..

[17] Pukala T.L., Brinkworth C.S., Carver J.A., Bowie J.H. (2004). Investigating the importance of the flexible hinge in caerin 1.1: Solution structures and activity of two synthetically modified caerin peptides. Biochemistry.

[18] Wang T., Andreazza H.J., Pukala T.L., Sherman P.J., Calabrese A.N., Bowie J.H. (2011). Histidine-containing host-defence skin peptides of anurans bind Cu^2+^. An electrospray ionisation mass spectrometry and computational modelling study. Rapid Commun. Mass Spectrom..

[19] Snyder L.A., Saunders N.J. (2006). The majority of genes in the pathogenic *Neisseria* species are present in the non-pathogenic *Neisseria lactamica*, including those designated as ‘virulence genes’. BMC Genom..

[20] Dover S.E., Aroutcheva A.A., Faro S., Chikindas M.L. (2008). Natural antimicrobials and their role in vaginal health: A short review. Int. J. Probiotics Prebiotics.

[21] Ravel J., Gajer P., Abdo Z., Schneider G.M., Koenig S.S.K., McCulle S.L., Karlebach S., Gorle R., Russell J., Tacket C.O. (2011). Vaginal microbiome of reproductive-age women. Proc. Natl. Acad. Sci. USA.

[22] Oakley B.B., Fiedler T.L., Marrazzo J.M., Fredricks D.N. (2008). Diversity of human vaginal bacterial communities and associations with clinically defined bacterial vaginosis. Appl. Environ. Microbiol..

[23] Srinivasan S., Liu C., Mitchell C.M., Fiedler T.L., Thomas K.K., Agnew K.J., Marrazzo J.M., Fredricks D.N. (2010). Temporal variability of human vaginal bacteria and relationship with bacterial vaginosis. PLoS ONE.

[24] McKinnon L.R., Achilles S.L., Bradshaw C.S., Burgener A., Crucitti T., Fredricks D.N., Jaspan H.B., Kaul R., Kaushic C., Klatt N. (2019). The evolving facets of bacterial vaginosis: Implications for HIV transmission. AIDS Res. Hum. Retrov..

[25] Tahara T., Kanatani K. (1997). Isolation and partial characterization of crispacin A, a cell-associated bacteriocin produced by Lacto bacillus crispatus JCM 2009. FEMS Microbiol. Lett..

[26] Stoyancheva G., Marzotto M., Dellaglio F., Torriani S. (2014). Bacteriocin production and gene sequencing analysis from vaginal *Lactobacillus* strains. Arch. Microbiol..

[27] Mokoena M.P. (2017). Lactic acid bacteria and their bacteriocins: Classification, biosynthesis, and applications against uropathogens: A mini-review. Molecules.

[28] Pusch O., Kalyanaraman R., Tucker L.D., Wells J.M., Ramratnam B., Boden D. (2006). An anti-HIV microbicide engineered in commensal bacteria: Secretion of HIV-1 fusion inhibitors by lactobacilli. AIDS.

[29] Lagenaur L.A., Sanders-Beer B.E., Brichacek B., Pal R., Liu X., Liu Y., Yu R., Venzon D., Lee P.P., Hamer D.H. (2011). Prevention of vaginal SHIV transmission in macaques by a live recombinant *Lactobacillus*. Mucosal Immunol..

[30] Zuend C.F., Nomellini J.F., Smit J., Horwitz M.S. (2019). A *Caulobacter crescentus* microbicide protects from vaginal infection with HIV-1JR-CSF in humanized bone marrow-liver-thymus mice. J. Virol..

[31] Pask J.D., Woodhams D.C., Rollins-Smith L.A. (2012). The ebb and flow of antimicrobial skin peptides defends northern leopard frogs (*Rana pipiens*) against chytridiomycosis. Global Change Biol..

[32] De Man J.D., Rogosa M., Sharpe M.E. (1960). A medium for the cultivation of *Lactobacilli*. J. Appl. Bacteriol..

[33] Cartwright C.P., Stock F., Gill V.J. (1994). Improved enrichment broth for cultivation of fastidious organisms. J. Clin. Microbiol..

